# The effect of acute respiratory distress syndrome on bone marrow-derived mesenchymal stem cells

**DOI:** 10.1186/s13287-018-0981-3

**Published:** 2018-09-26

**Authors:** Ben Antebi, Kerfoot P. Walker, Arezoo Mohammadipoor, Luis A. Rodriguez, Robbie K. Montgomery, Andriy I. Batchinsky, Leopoldo C. Cancio

**Affiliations:** 1 United States Army Institute of Surgical Research, San Antonio, TX USA; 2 Oak Ridge Institute for Science and Education, Oak Ridge, TN USA; 3 The Geneva Foundation, Tacoma, WA USA

**Keywords:** Bone marrow, Mesenchymal stem cells (MSCs), Acute respiratory distress syndrome (ARDS), Injury, Autologous therapy, Immunomodulation

## Abstract

**Background:**

It is known that, following a physiological insult, bone marrow-derived mesenchymal stem cells (MSCs) mobilize and home to the site of injury. However, the effect of injury on the function of endogenous MSCs is unknown. In this study, MSCs harvested from the bone marrow of swine with or without acute respiratory distress syndrome (ARDS) were assessed for their characteristics and therapeutic function.

**Methods:**

MSCs were harvested from three groups of anesthetized and mechanically ventilated swine (*n* = 3 in each group): 1) no ARDS (‘Uninjured’ group); 2) ARDS induced via smoke inhalation and 40% burn and treated with inhaled epinephrine (‘Injured Treated’ group); and 3) ARDS without treatment (‘Injured Untreated’ group). Cellular evaluation of the three groups included: flow cytometry for MSC markers; colony forming unit-fibroblast (CFU-F) assay; proliferative and metabolic capacity; gene expression using quantitative real-time polymerase chain reaction (qRT-PCR); and a lipopolysaccharide (LPS) challenge, with or without coculture with mononuclear cells (MNCs), for evaluation of their protein secretion profile using Multiplex. Statistical analysis was performed using one- or two-way analysis of variance (ANOVA) with a Tukey’s post-test; a *p*-value less than 0.05 was considered statistically significant.

**Results:**

Cells from all groups exhibited nearly 100% expression of MSC surface markers and retained their multidifferentiation capacity. However, the MSCs from the ‘Injured Untreated’ group generated a significantly higher number of colonies compared with the other two groups (*p* < 0.0001), indicative of increased clonogenic capacity following ARDS. Following an LPS challenge, the MSCs from the ‘Injured Untreated’ group exhibited a significant reduction in their proliferative capacity (*p* = 0.0002), significant downregulation in the expression of high-mobility group box 1 (HMGB1; *p* < 0.001), Toll-like receptor (TLR)-4 (*p* < 0.01), and vascular endothelial growth factor (VEGF; *p* < 0.05) genes, and significantly diminished secretory capacity for the inflammatory mediators interleukin (IL)-6 (*p* < 0.0001), IL-8 (*p* < 0.05), and tumor necrosis factor (TNF)-α (*p* < 0.05) compared with the ‘Uninjured’ group.

**Conclusions:**

The results suggest that, following ARDS, there is an increase in the clonogenic capacity of MSCs to increase the available stem cell pool in vivo. However, MSCs harvested from subjects with ARDS seem to exhibit a diminished capacity to proliferate, express regenerative signals, and secrete pro/anti-inflammatory mediators.

## Background

Over the last decade, mesenchymal stem cells (MSCs) have emerged as a potent therapeutic tool for treating acute respiratory distress syndrome (ARDS), as exemplified by their abundant use in numerous preclinical animal studies as well as early-phase clinical trials. The majority of clinical trials have focused on MSCs due to their immediate availability, proven safety, and robust regenerative potential. Emerging evidence indicates that MSCs exert their therapeutic effects predominantly via secretion of bioactive factors. Since the hallmark of ARDS is an acute inflammatory response, bioactive factors secreted by MSCs are expected to immuno-modulate the inflammatory response seen in ARDS. This inflammatory process can be modeled in vitro via exposure to lipopolysaccharide (LPS), an endotoxin secreted by gram-negative bacteria which mimics infection, the most common etiology of ARDS [1].

Typically, MSCs are obtained from either autologous or allogeneic sources. It is still unclear which source provides a superior regenerative response. For clinical studies, an allogeneic source is the most convenient as cells can be both expanded in vitro to attain a predetermined dose and fully characterized prior to administration. Indeed, as of July 2015, there were 374 clinical trials using MSCs for various indications, the majority of which were retrieved from allogeneic sources [2]. However, in critical care scenarios where treatment should be rendered as early as possible and when it cannot be planned in advance, autologous transplantation of the patient’s own stem cells may be the only viable choice. The autologous route may also be preferred in battlefield situations involving austere environments where allogeneic cell transplantation is logistically challenging. Additionally, autologous transplantation circumvents the risks and potential complications associated with allogeneic therapy, such as those occurring from a donor mismatch or disease transmission.

Autologous transplantation is not without its disadvantages. Aside from donor-site morbidity, one major yet understudied caveat of autologous stem cell transplantation is the unknown status of the cells. Typically, cases of autologous cell transplantation involve a patient who has an underlying chronic condition or who has undergone a traumatic injury. However, the effect of the condition or injury itself on the cell function is unknown. It is known that, following injury, MSCs are recruited from the bone marrow, mobilize to the systemic circulation, and home to the site of injury to initiate repair [3]. However, in cases involving an exacerbated inflammatory response, such as in ARDS, it is unknown whether MSCs are affected by the pathophysiology and its sequelae.

In this study, MSCs were harvested from the bone marrow of three groups of anesthetized and mechanically ventilated swine (*n* = 3 in each group): 1) no ARDS (‘Uninjured’); 2) ARDS induced via smoke inhalation and 40% burn and treated with inhaled epinephrine (‘Injured Treated’); and 3) ARDS without treatment (‘Injured Untreated’). We hypothesized that MSCs from injured swine possess an altered proliferative capacity and secretory signature compared with MSCs from uninjured swine. To investigate this, cells from all groups were characterized for the expression of common MSC surface markers using flow cytometry, multipotent capacity using differentiation assay, clonogenic abilities using a colony forming unit-fibroblast (CFU-F) assay, proliferative and metabolic capacity, gene expression using quantitative real-time polymerase chain reaction (qRT-PCR), and secretion profile using Multiplex before and after a LPS challenge.

## Methods

This study was approved by the US Army Institute of Surgical Research Animal Care and Use Committee. Research was conducted in compliance with the Animal Welfare Act, the implementing Animal Welfare Regulations, and the principles of the Guide for the Care and Use of Laboratory Animals, National Research Council. The facility’s Institutional Animal Care and Use Committee approved all research conducted in this study. The facility where this research was conducted is fully accredited by AAALAC International. Unless specified, all reagents were purchased from Thermo Fisher Scientific (Waltham, MA) for all in-vitro studies.

### Bone marrow aspiration procedure

Using a large ongoing study, bone marrow aspiration was performed on a total of nine Yorkshire female cross-bred swine (Midwest Research Swine, Gibbon, MN). Three of the animals were designated as donor swine for the retrieval of healthy cells, designated as the ‘Uninjured’ group. Six of the animals were exposed to smoke inhalation and large surface area burn to induce ARDS, as previously described [4]. Of those six, three animals were left untreated and designated as the ‘Injured Untreated’ group, while the other three swine were treated with inhaled racemic epinephrine and designated as the ‘Injured Treated’ group. Bone marrow was obtained at the end of each study after 48 h of mechanical ventilation and intensive care unit (ICU) management under full anesthesia, as previously described [5]. Briefly, swine were housed for at least 1 week to allow for acclimatization and testing for any pre-existing disease. Before aspiration, all materials were pre-coated with heparin to prevent clotting during collection and downstream processing. The area overlying the bone marrow site was aseptically prepared and a bone marrow aspiration needle (Arteriocyte Medical Systems, Hopkinton, MA) was drilled into the bone marrow compartment of the right or left iliac crest. Since a platelet concentration device (Magellan, Arteriocyte Medical Systems, Hopkinton, MA) was used to concentrate the bone marrow aspirate, in some instances peripheral blood was added to the bone marrow sample to satisfy the Magellan minimal volume requirement of 30 ml. Bone marrow aspirates were then filtered to remove residual fat, bone chips, and clots, after which the bone marrow aspirate was concentrated down approximately 10-fold using the Magellan.

### Mesenchymal stem cell culture

Concentrated bone marrow aspirates from which most of the red blood cells were removed were plated into standard cell-culture flasks in complete culture medium (CCM). The CCM consisted of minimal essential media-alpha formulation supplemented with 15% heat-inactivated, lot-selected, fetal bovine serum (Atlanta Biologicals, Atlanta, GA), 2 mM l-glutamine, and 1% antibiotic/antimycotic. The medium was changed every other day and the cells were allowed to grow for approximately 2 weeks. Once colonies formed, but before they overlapped, the plastic-adherent cells were enzymatically detached using 0.25% trypsin-EDTA and regarded as passage 0 (P0) MSCs. For all assays, P2–P3 MSCs were used and all experiments were performed in triplicate.

### Flow cytometry

Cells were examined by flow cytometry for the expression of the MSC markers CD90, CD105, and CD29, and for the absence of CD45. Antibodies used were FITC-conjugated mouse monoclonal CD45 (Bio-Rad, Hercules, CA), PE-conjugated mouse monoclonal CD105 (AbCam, Cambridge, MA), APC-conjugated mouse monoclonal CD90 (Abcam, Cambridge, MA), and APC-conjugated mouse monoclonal CD29 (BD Biosciences, San Jose, CA). To prevent nonspecific binding, 100 μl cell suspension at a concentration of 1 × 10^6^/ml were incubated for 5 min with 1% bovine serum albumin. Antibodies were then added followed by incubation for 15 min at 22 °C followed by a phosphate-buffered saline (PBS) wash step. Analyses were carried out on a BD FACSCanto II using the BDFACS Diva software.

### Multipotent differentiation capacity

A multidifferentiation assay was used to evaluate the multipotent capacity of MSCs to give rise to osteoblasts, adipocytes, and chondrocytes using a commercially available differentiation medium (StemPro Differentiation Kits, Thermo Fisher Scientific) according to the manufacturer’s instructions. For this purpose, the MSCs from the different groups were cultured in eight-well chamber slides for histological evaluation as well as on six-well plates for gene expression assessment using qRT-PCR. Briefly, for osteogenic differentiation, cells were cultured in osteogenic differentiation medium. The differentiation medium was replaced twice weekly. After 21 days, differentiation was assessed by quantifying calcium deposits using Alizarin Red staining and by assessing osteogenic differentiation genes using qRT-PCR. For adipogenic differentiation, cells were cultured in adipogenic differentiation medium. After 14 days, adipogenic differentiation was assessed by Oil Red O staining of lipid vacuoles and by assessing adipogenic differentiation genes using qRT-PCR. For chondrogenic differentiation, 5-μl droplets of cell solution at a density of 1.6 × 10^7^ cells/ml was seeded in the center of a multi-well plate to form a micromass cultured for 2 h, and then induced using the chondrogenic differentiation medium. Differentiation medium was changed every other day. After 14 days, chondrogenic differentiation was assessed by Alcian Blue staining of sulfated proteoglycans.

### Colony-forming unit fibroblast assay

The CFU-F assay was used as an indicator of progenitor cells, as previously described [6]. Briefly, passage 2 MSCs were plated at 100 and 200 cells/well on six-well plates in a total of 3 ml CCM per well. Medium was changed every 3–4 days and the cells were allowed to grow for 7–10 days. Prior to the overlap of colonies, cells were washed with PBS and fixed with chilled methanol for 10 min at room temperature. Next, the plates were allowed to air dry and stained with Giemsa to allow for visualization. Colonies larger than 50 cells were enumerated and reported as CFUs/well.

### Cell proliferation and metabolic activity

MSCs from the three different groups were evaluated for their metabolic activity using the Vybrant assay (Thermo Fisher Scientific, Waltham, MA) according to the manufacturer’s instructions. In this assay, nonfluorescent resazurin (R-12204) is reduced by viable cells to red-fluorescent resorufin during a 15-min incubation period. The reaction product exhibits absorption/emission at wavelengths of 563/587 nm, which were detected using a SpectraMax i3X system (Molecular Probes). To perform this, MSCs were seeded at 1000 cells/cm in triplicate and their medium was evaluated along three different time points on days 3, 7, and 10. For analysis, the metabolic activity was normalized to DNA quantity in the wells to evaluate metabolism on a cellular level (i.e., irrespective of cell number).

Following the metabolic assay, the MSCs from the different groups were placed in cell lysis buffer (Cell Signaling Technology, Danvers, Massachusetts). Following lysis at each time point, the multiwell plates were stored at −80 °C until batch analysis. Next, plates were thawed and DNA concentration was measured using the Quant-iT PicoGreen assay (Invitrogen) to evaluate cell proliferation on days 3, 7, and 10, as previously described [6]. Briefly, an ultrasensitive fluorescent nucleic acid stain was used to quantify double-stranded DNA in solution. Samples were prepared by diluting with 1× TE buffer (1:100) then plated in duplicate. PicoGreen working solution was then added to prediluted samples. Plates were run on a SpectraMax i3X system (Molecular Probes) and fluorescence measured at a wavelength of 502/523 nm. For qualitative assessment of proliferation, MSCs were stained with a fluorescent Live/Dead Cell Viability Kit, according to the manufacturer’s instruction (Life Technologies, Grand Island, NY) and as previously described [7]. In this live/dead assay, the cytoplasm of viable cells is stained green (excitation/emission 495/515 nm) and the nucleus of dead cells is stained orange (excitation/emission 528/617 nm). If dead cells were present, the live/dead images were overlaid for analysis.

### Quantitative real-time polymerase chain reaction

To determine gene expression via qRT-PCR, total RNA was extracted from MSCs using Trizol (Thermo Fisher Scientific) and reverse-transcribed using a High Capacity cDNA Archive Kit (Applied Biosystems, Foster City, CA). The transcripts of interest were amplified from cDNA using Taqman Universal PCR Master Mix and all the primers were purchased from Applied Biosystems. Amplification and detection were carried out with a StepOnePlus Real-Time PCR System (Applied Biosystems) for high-mobility group box 1 (HMGB1), vascular endothelial growth factor (VEGF), Toll-like receptor (TLR)-4, angiopoietin 1 (Ang-1), and sex determining region Y-box 2 (SOX-2). For normalization, MSCs prior to LPS exposure were used as reference samples. For differentiation, osteocalcin (BGLAP), osteonectin (SPARC), and osteopontin (SPP1) genes were used to assess osteogenic differentiation, whereas lipoprotein lipase (LPL) and peroxisome proliferator-activated receptor gamma (PPARγ) genes were used to evaluate adipogenic differentiation. For normalization, MSCs maintained in regular CCM for 14 and 21 days were used as reference samples. Beta-actin was used as the housekeeping gene. All assays were performed in duplicate and gene expression is expressed as a relative quotient (RQ) calculated from ΔΔCt of the sample of interest, where C_T_ is the threshold cycle.

$$ \Delta {C}_{T- Gene\ of\ Interest}={C}_{T- Housekeeping-{C}_{T- Gene\ of\ Interest}} $$

$$ \Delta \Delta {C}_{T- Gene\ of\ Interest}=\Delta {C}_{T- Gene\ of\ Interest}-\Delta {C}_{T- Reference} $$

$$ RQ={2}^{-\Delta \Delta {C}_{T- Gene\ of\ Interest}} $$

### Protein measurements

The secretory profile of the different MSCs was assessed before and after LPS treatment using either the cytokine-chemokine 5-plex or 13-plex kit (Millipore, Billerica, MA) to evaluate granulocyte-macrophage colony stimulating factor (GM-CSF), interferon (IFN)-γ, tumor necrosis factor (TNF)-α, and interleukin (IL)-1α, IL-1β, IL-1ra, IL-2, IL-4, IL-6, IL-8, IL-10, IL-12, and IL-18. To accomplish this, the supernatants of samples were spun down to remove any remaining cells and stored at −80 °C until simultaneous analysis. Samples were run on a BioPlex 200 system (Bio-Rad, Hercules, CA; http://www.bio-rad.com) following the manufacturer’s instructions. Briefly, 25 μl of the samples were applied in duplicate to the appropriate wells. Specific antibody-coated beads corresponding to the above cytokines were added to the wells and incubated for approximately 18 h at 4 °C on an orbital shaker at approximately 700 rpm. The wells were then washed, biotinylated antibodies were added, and the mixture was incubated at room temperature for 2 h while shaking. Next, streptavidin-phycoerythrin was added and the wells were incubated for 30 min at room temperature while shaking to detect the cytokine-antibody complexes. The concentration of analytes was determined based on the spectral properties of the beads and the R-phycoerythrin fluorescence. Data were analyzed using Bio-Rad BioPlex Manager software, version 6.0, and standardized to the total amount of protein. For total protein, the Pierce™ 660 nm protein assay (Thermo Scientific) was used according to the manufacturer’s instructions. Protein concentrations are reported as nanograms per gram of total protein (ng/gTP).

### Lipopolysaccharide challenge with or without mononuclear cell coculture

Porcine MSCs were plated at a density of 50,000 cells per well in 24-well plates in CCM for 2–3 h to allow for initial cell attachment. In one experiment, MSCs were directly challenged with 1 μg/ml LPS for overnight culture. In a second experiment, 500,000 mononuclear cells (MNCs) were added to the wells followed by a challenge with LPS for overnight culture. In a separate set of coculture preparations, the proliferative and metabolic response was also assessed before and after the addition of LPS and fluorescent live/dead images were acquired. In both experiments (i.e., MSCs + LPS and MSCs + MNCs + LPS), the medium was collected after 24 h of LPS addition and analyzed for protein content using both total protein and the cytokine-chemokine 13-plex kit (Millipore, Billerica, MA).

### Statistical analysis

Results are presented as means ± standard errors of the mean (SEM). All statistical tests were performed with the aid of GraphPad Prism version 7.01. A one- or two-way analysis of variance (ANOVA) was used followed by a Tukey’s multiple comparisons post-test; a *p* value less than 0.05 was considered statistically significant.

## Results

### Functional characteristics of MSCs

**Fig. 1:**
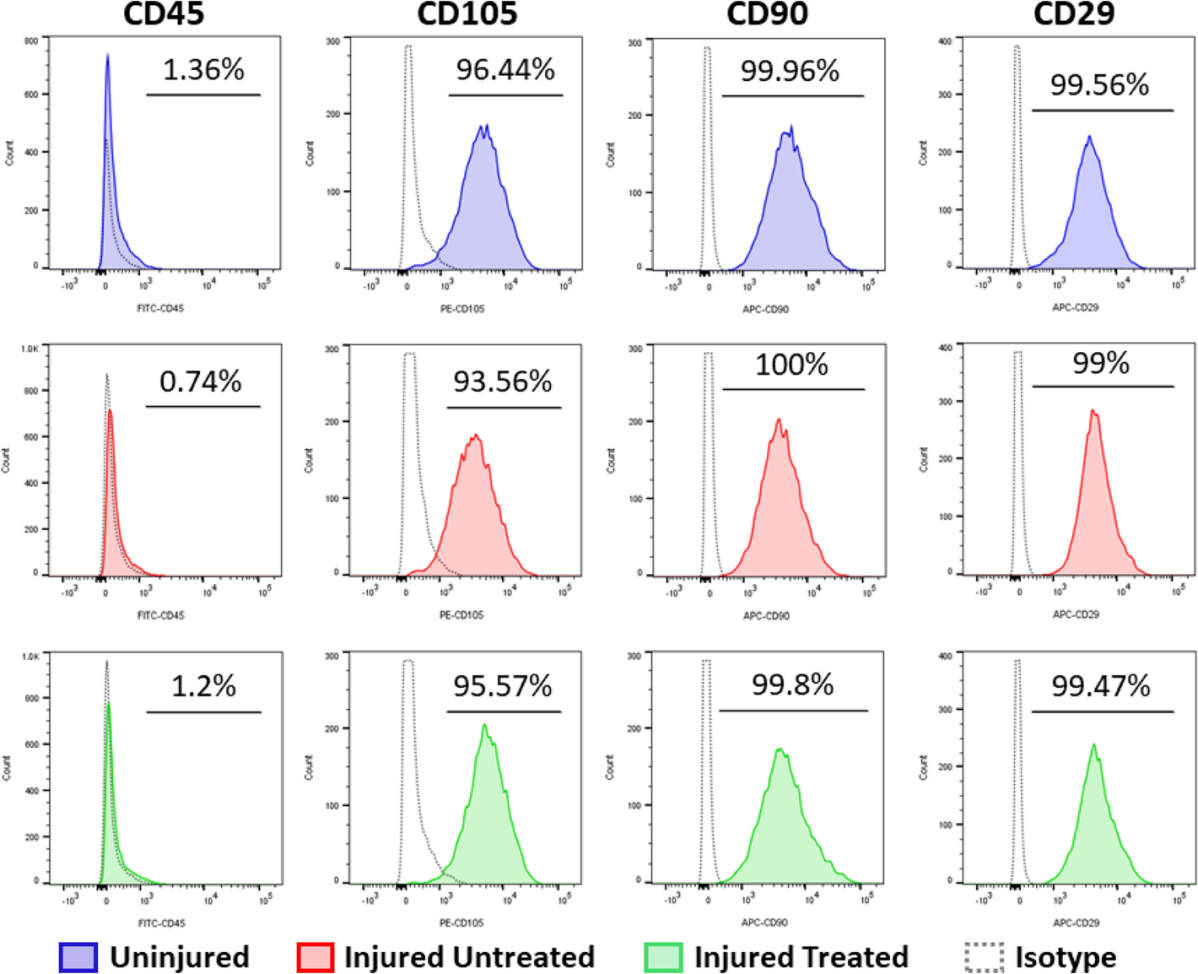
Surface marker expression of MSCs from different groups of swine: ‘Uninjured’; ‘Injured Untreated’; and ‘Injured Treated’. Cells expressed nearly 100% of common MSC surface markers, including CD29 (99.56 ± 0.23% (mean ± SEM) for ‘Uninjured’; 99 ± 0.64% for ‘Injured Untreated’; 99.47 ± 0.43% for ‘Injured Treated’), CD90 (99.96 ± 0.02% for ‘Uninjured; 100 ± 0.0% for ‘Injured Untreated’; 99.8 ± 0.13% for ‘Injured Treated’), CD105 (96.44 ± 0.92% for ‘Uninjured; 93.56 ± 2.03% for ‘Injured Untreated’; 95.57 ± 2.14% for ‘Injured Treated’), and a lack of expression of CD45 (1.36 ± 0.48% for ‘Uninjured; 0.74 ± 0.09% for ‘Injured Untreated’; 1.2 ± 0.18% for ‘Injured Treated’)

**Fig. 2:**
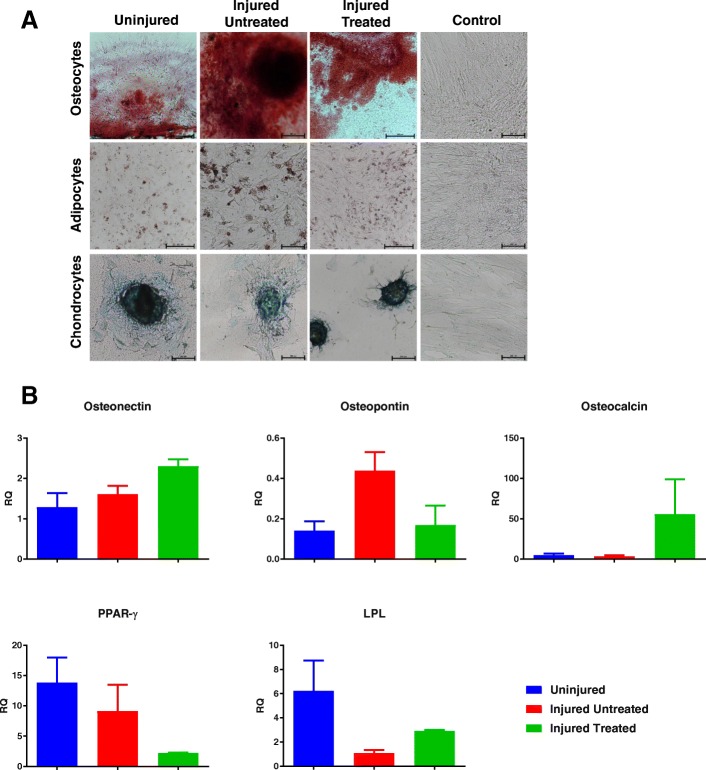
Multidifferentiation capacity of MSCs from the three groups of swine. **a** Histological images demonstrate that MSCs from all groups possess multidifferentiation capacity, exemplified by the ability to differentiate to osteocytes, adipocytes, and chondrocytes in vitro; scale bars = 250 μm. **b** Gene expression analysis revealed no significant differences in the ability of MSCs to differentiate down the osteogenic (osteonectin, osteopontin, and osteocalcin genes) and adipogenic (peroxisome proliferator-activated receptor (PPAR)-γ and lipoprotein lipase (LPL) genes) lineages. RQ relative quotient

The effect of ARDS on the characteristics and function of bone marrow MSCs was evaluated using a panel of in-vitro assays. According to the International Society of Cellular Therapy (ISCT), the minimal criteria to define MSCs are: adherence to plastic; expression of specific surface markers; and the ability to differentiate to osteocytes, adipocytes, and chondrocytes in vitro [8]*.* First, flow cytometry evaluation showed that, despite injury, cells from all groups exhibited nearly 100% expression of MSC surface markers (Fig. 1). Similarly, MSCs from all groups possessed multipotent capacity, demonstrated by the ability to differentiate down the osteogenic, adipogenic, and chondrogenic lineages as seen from histological observations (Fig. 2a). To verify the histological data, quantitative gene expression following differentiation was carried out. No differences were observed in the capacity of the MSCs from the different groups to differentiate down the osteogenic and adipogenic lineages (Fig. 2b), thus satisfying the ISCT criteria for defining MSCs.

**Fig. 3:**
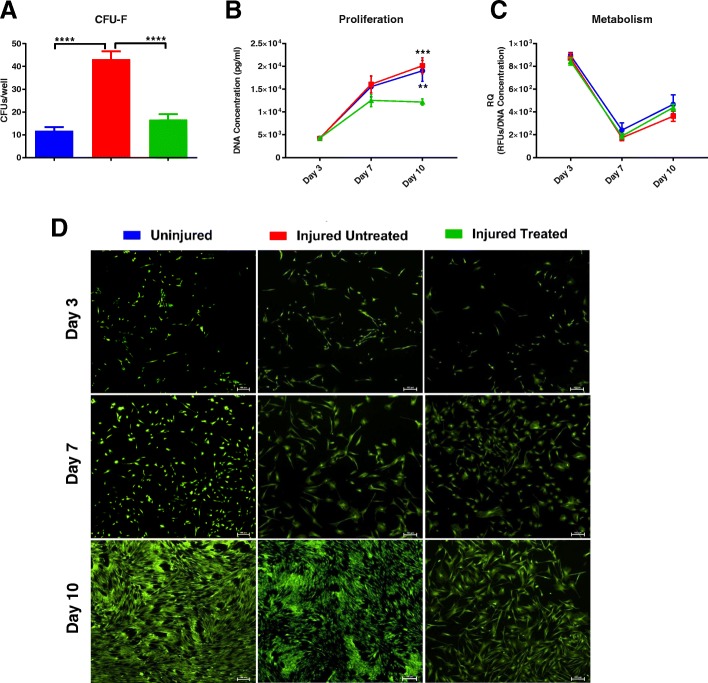
Functional characteristics of MSCs from the different groups. **a** Clonogenic capacity of MSCs from the ‘Injured Untreated’ group as measured by the colony-forming unit fibroblast (CFU-F) assay was significantly higher than the other two groups. **b** Proliferation of MSCs from the ‘Uninjured’ and ‘Injured Untreated’ groups was significantly higher than MSCs from the ‘Injured Treated’ group (*p* < 0.01 and *p* < 0.001, respectively). **c** No differences were observed in the metabolic activity of the MSCs between the different groups. **d** Representative images of proliferation of the three groups of MSCs is shown throughout the 10-day study period through fluorescent staining of the cytoplasm of viable cells; scale bars = 100 μm. **p < 0.01, ***p < 0.001, ****p < 0.0001. RFUs - relative fluorescent units , RQ - relative quotient

The CFU-F assay was used to evaluate the clonogenic capacity of MSCs, which is another central feature of their therapeutic function. In contrast to the unaltered surface marker expression and multipotent differentiation capacity, the MSCs from the ‘Injured Untreated’ group generated a significantly higher number of colonies compared with the other two groups (*p* < 0.0001), indicative of increased clonogenic capacity (Fig. 3a). Similarly, the proliferation (Fig. 3b) of MSCs from the ‘Injured Untreated’ group was significantly higher than the ‘Injured Treated’ group (*p* < 0.001), but was similar to the ‘Uninjured’ group. No differences were detected among the groups regarding their metabolic activity (Fig. 3c). Fluorescent live/dead imaging of the MSCs from the different groups along the 10-day study further corroborated the quantitative proliferation data (Fig. 3d).

### MSC function following LPS treatment

Since it is now well established that the predominant mechanism by which MSCs elicit their therapeutic response in the setting of ARDS is via secretion of bioactive products [1], we wanted to evaluate their secretion profile with and without the addition of LPS. The addition of LPS served to mimic infection, which is a common sequela of ARDS following smoke inhalation and burn. This allowed us to evaluate MSC function in response to a bacterial endotoxin.

**Fig. 4:**
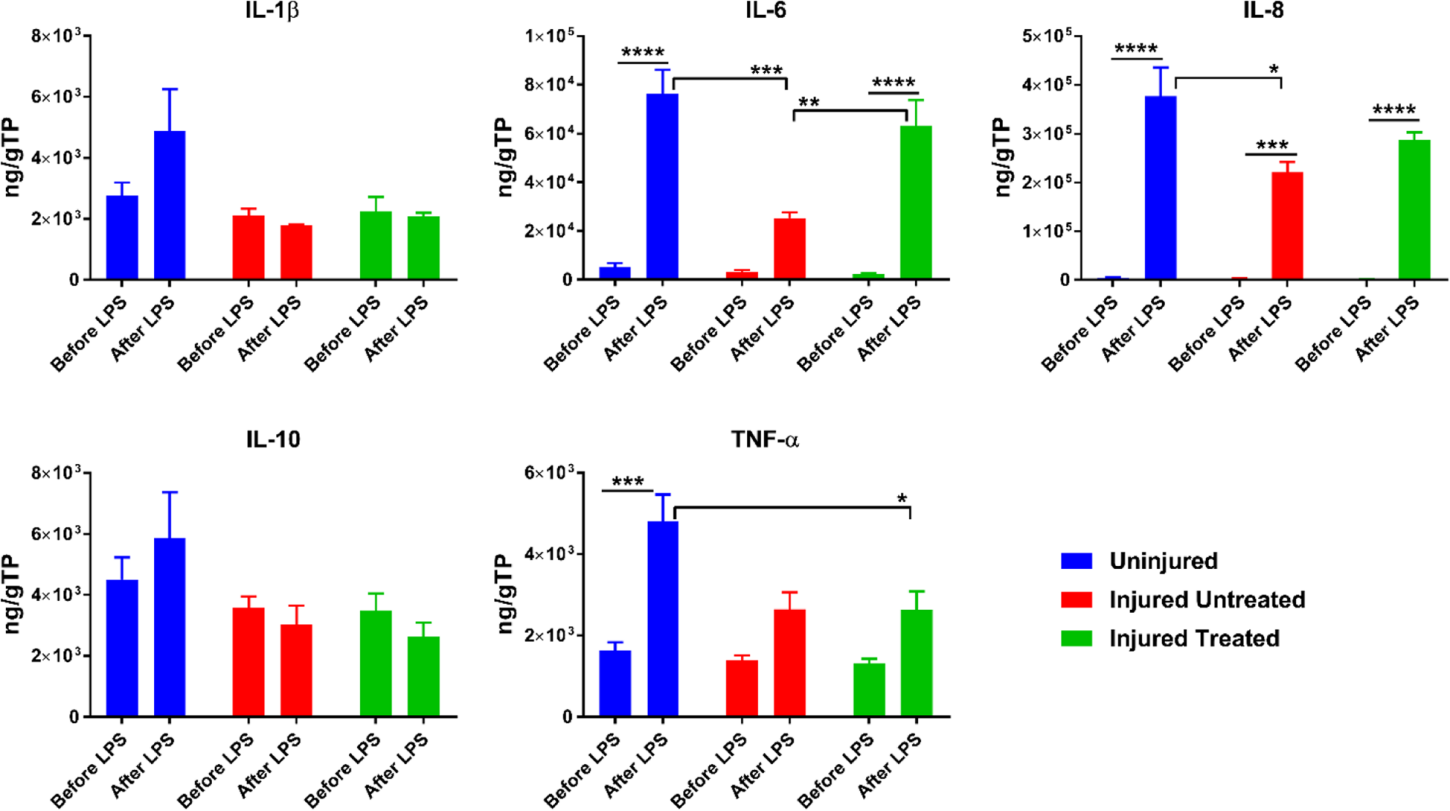
Secretion profile of MSCs from the different groups before and after LPS treatment. MSCs from the ‘Injured’ groups demonstrated diminished capacity to secrete interleukin (IL)-6, IL-8, and tumor necrosis factor (TNF)-α compared with ‘Uninjured’ MSCs. Within each group, lipopolysaccharide (LPS) exposure induced significant upregulation of the inflammatory markers, which was most pronounced in the ‘Uninjured’ MSCs. *p < 0.05, **p < 0.01, ***p < 0.001, **** *p* < 0.0001. TP total protein

Before LPS treatment, MSCs from the ‘Uninjured’ group exhibited a more potent secretion of pro/anti-inflammatory mediators with a significant secretion of IL-8 (*p* < 0.05) compared with the ‘Injured Treated’ group. Following LPS challenge, this phenomenon was even more pronounced, demonstrated by significant secretion in IL-6 (*p* < 0.001), IL-8 (*p* < 0.05), and TNF-α (*p* < 0.05) compared with the ‘Injured’ groups. Evaluation of the secreted factors within each group after LPS treatment revealed a significant increase in IL-6 from ‘Uninjured’ and ‘Injured Treated’ MSCs (*p* < 0.0001), IL-8 from all MSCs (*p* < 0.001), and TNF-α from ‘Uninjured’ MSCs (*p* < 0.001) (Fig. 4).

**Fig. 5:**
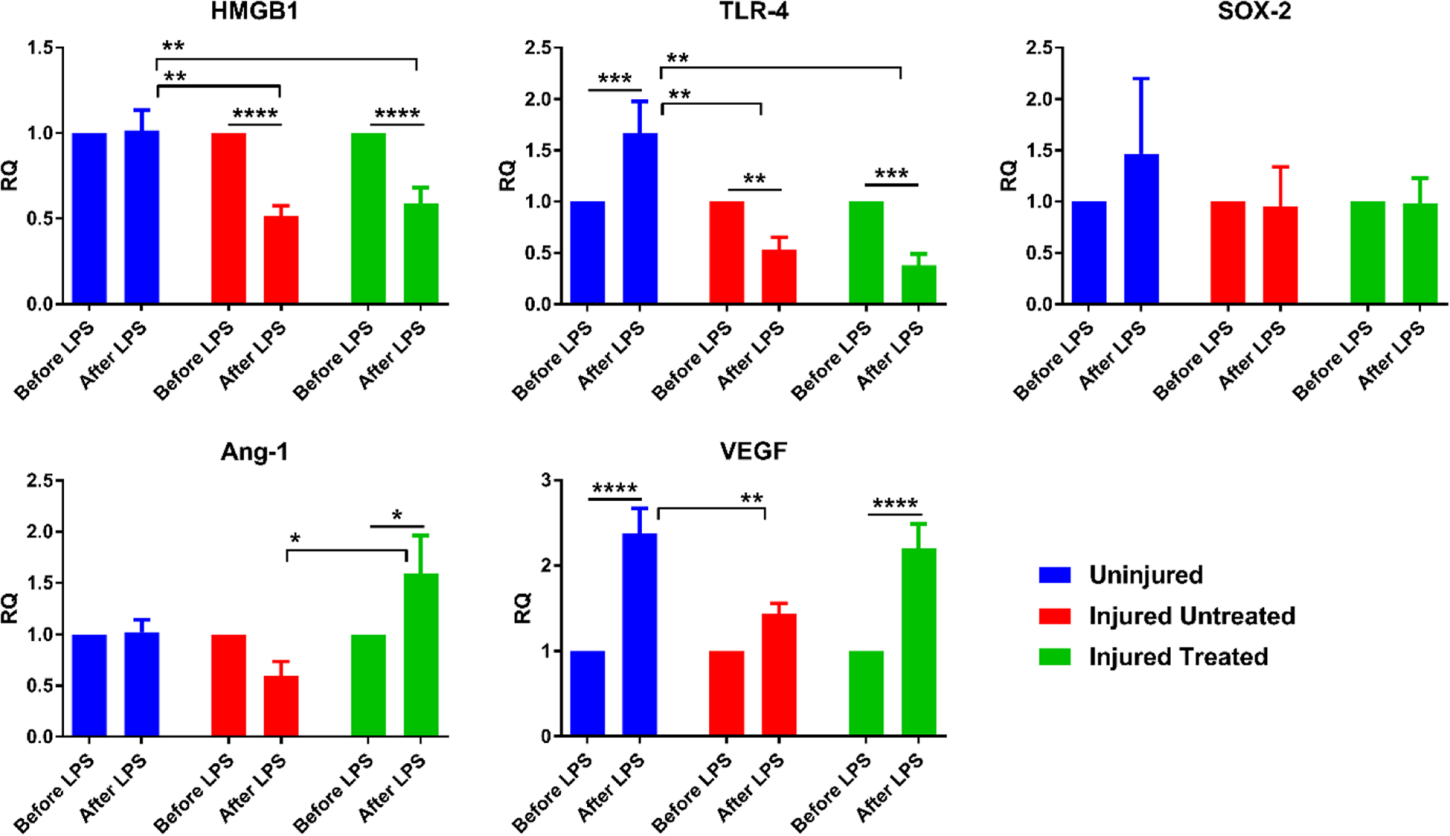
Gene expression of MSCs from the different groups after LPS treatment. Significant downregulation of high-mobility group box 1 (HMGB1) and Toll-like receptor (TLR)-4 genes in ‘Injured’ MSCs compared with ‘Uninjured’ MSCs (*p* < 0.01). Additionally, downregulation of angiopoietin 1 (Ang-1) and vascular endothelial growth factor (VEGF) genes in ‘Injured Untreated’ MSCs compared with ‘Injured Treated’ and ‘Uninjured’ MSCs, respectively (*p* < 0.05). Within each group, lipopolysaccharide (LPS) exposure induced significant downregulation of HMGB1 (*p* < 0.0001) and TLR-4 (*p* < 0.01) genes in ‘Injured’ MSC compared with upregulation in ‘Uninjured’ MSCs. For angiogenic genes, VEGF was upregulated in both ‘Uninjured’ and ‘Injured Treated’ MSCs (*p* < 0.0001) while Ang-1 was upregulated in ‘Injured Treated’ MSCs only (*p* < 0.05). *p < 0.05, **p < 0.01, ***p < 0.001, ****p < 0.0001. RQ relative quotient, SOX-2 sex determining region Y-box 2

In addition to their secretion profile, we also assessed the gene expression in response to LPS. TLR-4 is the receptor for LPS as well as one of the key receptors for HMGB1. HMGB1 is a protein that is secreted by various immune cells, including MSCs, in response to tissue damage and inflammation, the hallmark of ARDS. We found significant downregulation in the expression of HMGB1 and TLR-4 genes in ‘Injured’ MSCs compared with ‘Uninjured’ MSCs (*p* < 0.01). To evaluate the effects of ARDS on the angiogenic properties of MSCs, the VEGF and Ang-1 genes were evaluated. We observed a significant downregulation of Ang-1 (*p* < 0.05) and VEGF (*p* < 0.01) in ‘Injured Untreated’ MSCs compared with ‘Injured Treated’ and ‘Uninjured’ MSCs, respectively. With regard to ‘stemness’, no significant differences were found in the expression of the stem cell gene SOX-2. Assessment of gene expression within each group after LPS exposure revealed a significant downregulation in HMGB1 (*p* < 0.0001) and TLR-4 (*p* < 0.01) in ‘Injured’ MSCs. Conversely, in ‘Uninjured’ MSCs, significant upregulation of TLR-4 (*p* < 0.001) and VEGF (*p* < 0.0001) genes was observed, while in ‘Injured Treated’ MSCs the angiogenic genes Ang-1 and VEGF were significantly upregulated (*p* < 0.05 and *p* < 0.0001, respectively) after LPS exposure (Fig. 5).

### MSC coculture with MNCs

**Fig. 6:**
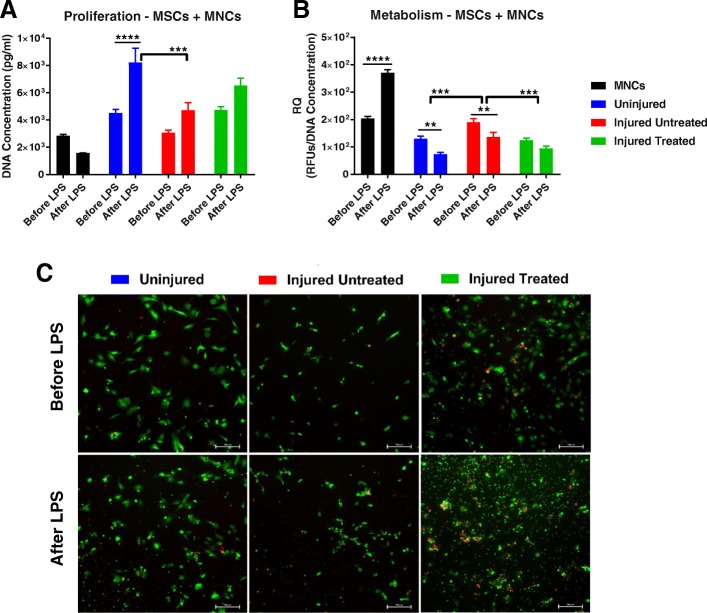
Proliferation and metabolic activity of MSCs cocultured with MNCs before and after LPS treatment. **a** After lipopolysaccharide (LPS) exposure, there was an increase in proliferation in the cocultures with mesenchymal stem cells (MSCs) and cell death in the mononuclear cell (MNC) alone group; the ‘Uninjured’ group generated significantly more cells than the ‘Injured Untreated’ group (*p* < 0.001). Additionally, in the ‘Uninjured’ group, significantly more cells were generated after LPS exposure, whereas no differences were detected among the ‘Injured’ MSCs. **b** The metabolic activity was significantly higher (before and after LPS exposure) in ‘Injured Untreated’ MSCs (*p* < 0.001). Within each group, a significant increase in the metabolic activity of MNCs was observed after LPS exposure (*p* < 0.0001), while the metabolic activity was significantly decreased in ‘Uninjured’ and ‘Injured Untreated’ cocultures. **c** Overlaid fluorescent live/dead images showing viable cytoplasm in green, while the nuclei of dead cells are stained red. Increased cell death is seen following LPS exposures in all groups; scale bars = 500 μm. *p < 0.05, **p < 0.01, ***p < 0.001, ****p < 0.0001. Relative quotient

To gain an insight into MSC immunomodulatory function as seen in vivo, their proliferation, metabolic activity, and secretion profile was also examined before and after LPS challenge in a coculture system with MNCs. Prior to the addition of LPS, no significant differences in proliferation or metabolic activity were observed. However, following treatment with LPS, the ‘Uninjured’ group had significantly greater cell yield than before LPS exposure (*p* < 0.0001) and as compared with the ‘Injured Untreated’ group (*p* < 0.001). Exposure to LPS appeared to induce cell death in MNCs alone, whereas when cocultured with MSCs an increase in proliferation was seen (Fig. 6a). When normalized to cell number, the metabolic activity of the MSCs cocultured with MNCs was significantly different among the three groups, with a significant increase (*p* < 0.001) in the ‘Injured Untreated’ group, both before and after LPS exposure. Examining the effect within each group revealed a significant decrease (*p* < 0.01) in the metabolic activity of cocultured MSCs from the ‘Uninjured’ and ‘Injured Treated’ groups and a significant increase (*p* < 0.0001) in MNCs alone after the addition of LPS (Fig. 6b). Overlaid, fluorescent live/dead images demonstrated a degree of cell death in MNCs after the addition of LPS, which further corroborated the quantitative data (Fig. 6c).

**Fig. 7:**
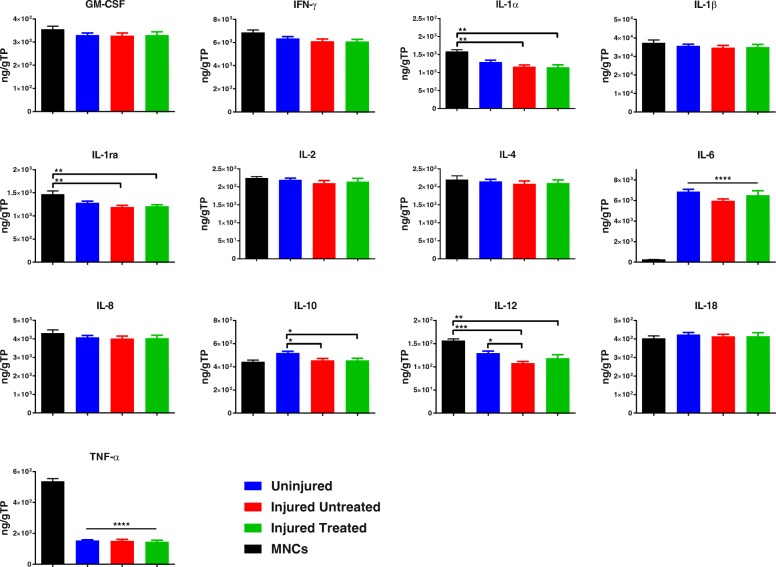
Secretion profile of MSCs cocultured with MNCs in response to LPS treatment. ‘Injured’ MSCs exhibit diminished capacity to secrete interleukin (IL)-1α and IL-1ra compared with mononuclear cells (MNCs) (*p* < 0.01) as well as reduced capacity to secrete IL-10 and IL-12 compared with ‘Uninjured’ MSCs (*p* < 0.05); all MSCs were able to significantly both secrete high levels of IL-6 and suppress tumor necrosis factor (TNF)-α production compared with MNCs alone (*p* < 0.0001). *p < 0.05, **p < 0.01, ***p < 0.001, ****p < 0.0001. TP total protein

With regard to their secretion profile, the ‘Injured’ MSCs had reduced levels of the proinflammatory cytokines IL-1α and IL-12 as well as the anti-inflammatory cytokines IL-1ra and IL-10. IL-6 levels were also significantly higher (*p* < 0.0001) in all MSC groups compared with secretion from MNCs alone. However, irrespective of the injury, MSCs from the three groups were able to significantly suppress (*p* < 0.0001) the secretion of the proinflammatory cytokine TNF-α compared with MNCs alone (Fig. 7).

## Discussion

Over the last decade, MSCs have emerged as potent therapeutic candidates for treating a large array of conditions. Recent preclinical data and early-phase clinical studies suggest that MSCs can mitigate ARDS when delivered as an allogeneic therapy. However, little is known about autologous stem cell therapy, particularly for patients presenting with ARDS. In this study, we harvested MSCs from the bone marrow of three groups of animals: 1) Uninjured swine; 2) swine with ARDS that were untreated; and 3) swine with ARDS that were treated with inhaled epinephrine. Following isolation, the MSCs were evaluated for their characteristics and function to assess the effect of ARDS, due to smoke inhalation and burn, on MSCs.

We first observed that, despite the injury, MSCs from all groups exhibited over 95% expression of common MSC surface markers (Fig. 1). Moreover, irrespective of their group, all MSCs maintained their multipotent differentiation potential, demonstrated by their ability to give rise to osteocytes, adipocytes, and chondrocytes in vitro (Fig. 2a, b). It is important to note that, although no significant differences were observed in terms of gene expression following induction of differentiation, there was an upregulation in osteopontin in ‘Injured Untreated’ MSCs. Aside from its role in biomineralization, osteopontin is a master regulator during inflammation, and is shown to be activated by various bioactive factors (such as LPS, IFN-γ, TNF-α, and transforming growth factor (TGF)-β) [9]. Therefore, since other osteogenic genes were not concomitantly upregulated in the ‘Injured Untreated’ MSCs, we suggest that its upregulation is due to an internal inflammatory cell process. This, however, needs to be further evaluated. In comparison, an upregulation was observed in the adipogenic genes of ‘Uninjured’ MSCs, suggesting an adipogenic predisposition of the porcine bone marrow MSCs which is later diminished by the injury. Chinnadurai et al. isolated bone marrow MSCs from Crohn’s patients. Similar to our results, they found no differences in the expression of MSC surface markers; however, their differentiation potential was not evaluated. They concluded that MSCs from Crohn’s patients are functionally analogous to healthy MSCs following IFN-γ prelicensing [10].

In addition to their differentiation potential and surface marker expression, we assessed their clonogenic, proliferative, and metabolic capacity. Interestingly, we observed a significant increase in the clonogenic ability of MSCs that were harvested from the ‘Injured Untreated’ group (Fig. 3a). Although this needs to be further elucidated, increased clonogenic capacity may suggest that, following smoke inhalation and burn, MSCs may be triggered to undergo self-renewal to increase the available stem cell pool in the bone marrow. The increased proliferative activity of the ‘Injured Untreated’ MSCs, as compared with ‘Injured Treated’ MSCs, further substantiates these findings (Fig. 3b). Interestingly, we observed a decrease in the metabolic activity of MSCs from all groups with a concomitant increase in cell proliferation on day 7 (Fig. 3c). The same inverse relationship was observed when MSCs were cocultured with MNCs (Fig. 6a, b). It is known that metabolic activity does not necessarily correlate with cell proliferation [11] and, in certain instances, may be inversely correlated. For example, MSCs that undergo stress, such as ischemia, may exhibit a metabolic switch of function towards survival with increased metabolism and decreased proliferative rates [12]. Silva et al. evaluated bone marrow MNCs from healthy mice and mice with ARDS induced via LPS. Similar to our results, they reported higher number of colonies (via a CFU-F assay) as well as a shorter doubling time in bone marrow cells from injured mice. They concluded that although the ‘Injured’ MNCs had different characteristics, they were as effective as healthy MNCs in reducing inflammation and tissue remodeling [13].

Since it is now well established that secretion of bioactive factors is the predominant mechanism by which MSCs exert their therapeutic function, we evaluated the secretion of various inflammatory mediators before and after exposure to LPS. Our results demonstrate that, before exposure to LPS, the ‘Injured’ MSCs had secreted lower levels of various pro- and anti-inflammatory cytokines compared with ‘Uninjured’ MSCs. Intriguingly, following exposure to LPS, this trend was even more pronounced, with significantly diminished levels of the proinflammatory cytokines IL-6 (*p* < 0.001), IL-8 (*p* < 0.05), and TNF-α (*p* < 0.05) compared with ‘Uninjured’ MSCs. The diminished capacity of ‘Injured’ MSCs to stimuli was further evident by evaluating the response to LPS within each group. Following LPS exposure, the ‘Uninjured’ MSCs triggered a significant increase in secretion of various proinflammatory cytokines, particularly TNF-α, compared with ‘Injured’ MSCs. It may be argued that the diminished capacity to secrete inflammatory mediators from ‘Injured’ MSCs may be physiologically beneficial; however, levels of the anti-inflammatory IL-10 were also reduced, albeit not significantly (*p* < 0.09), from ‘Injured’ MSCs. Diaz de la Guardia et al. performed detailed characterization of MSCs from patients with acute myeloid leukemia (AML). Similar to our results, they observed increased clonogenic capacity from MSCs derived from AML patients. Additionally, the MSCs maintained their differentiation potential and exhibited diminished secretory capacity of various proinflammatory cytokines. They concluded that, although AML-derived MSCs showed functional differences from healthy MSCs, they maintained a similar (undesired) capacity to protect leukemic cells from chemotherapy [14].

Similarly, when we looked at the gene level, we found downregulation of the genes HMGB1 and one of its key receptors, TLR-4, from the ‘Injured’ MSCs. Tamai et al. showed that HMGB1 plays an instrumental role in triggering the mobilization of MSCs from the bone marrow to sites of epithelial injury [15]. Additionally, Hayakawa et al. demonstrated that HMGB1 can enhance stem cell recruitment and plasticity, proliferation, and differentiation within the damaged brain after stroke [16]. As expected, TLR-4, the receptor for LPS, was upregulated following exposure to LPS in the ‘Uninjured’ group compared with both ‘Injured’ groups (*p* < 0.01). Similarly, both the VEGF and Ang-1 genes were downregulated in ‘Injured untreated’ MSCs. The VEGF and Ang-1 proteins play a pivotal role in angiogenesis, and specifically in the repair of the endothelial/epithelial membrane following ARDS by stimulating the migration and proliferation of endothelial cells [17–19]. Therefore, downregulation of these key genes may be detrimental to the overall function of MSCs. Administration of nebulized epinephrine has previously been shown to significantly improve pulmonary gas exchange and limit the pulmonary vascular hyperpermeability in a sheep model of ARDS induced by smoke inhalation and burn [20]. These findings may explain the upregulation of VEGF and Ang-1 genes in MSCs isolated from ‘Injured Treated’ animals, since the extent of hyperpermeability depends on the integrity of the endothelial/epithelial membrane. Thus, upregulation of these key genes in endogenous cells may account for part of the mechanisms involved in the repair of the endothelial membrane and overall attenuation of lung injury. These data are further substantiated by evaluating gene responses within each group. Both TLR-4 (*p* < 0.01) and HMGB1 (*p* < 0.0001) are significantly downregulated following LPS exposure in ‘Injured’ MSCs, whereas in ‘Uninjured’ MSCs HMGB1 remains unchanged and TLR-4 is significantly upregulated (*p* < 0.001). This suggests that TLR-4 may play a pivotal role in MSC response to injury, which is further supported by the diminished secretion of cytokines from ‘Injured’ cells. TLR-4 is one of the main receptors for LPS, which activates the transcription factor NF-κβ to upregulate the production of proinflammatory cytokines such as TNF-α, IL-6, and IL-8, as well as anti-inflammatory cytokines such as IL-10 [14].

Next, we evaluated the effects of LPS on MSCs when cocultured with MNCs to gain an insight into the immunomodulatory function of MSCs as seen in vivo. In terms of proliferation, the ‘Injured’ MSCs had significantly less proliferation than ‘Uninjured’ MSCs following exposure to LPS. An increased proliferative capacity of MSCs following stimulation with LPS is well documented in the literature [21–23]. Whether differences in cell yield stem from an increase in the proliferation of either MSCs or MNCs, it appears that the ‘Injured’ MSCs are unable to produce the same beneficial response as the ‘Uninjured’ MSCs. Additionally, and importantly, following exposure to LPS the MSCs from all groups, but significantly more from the ‘Uninjured’ group, appeared to exhibit a cytoprotective effect since the MNCs experienced cell death when cultured alone (Fig. 6a).

With regard to the secreted factors in this coculture platform, significantly decreased levels of the proinflammatory cytokine IL-1α (*p* < 0.01) and its receptor the anti-inflammatory IL-1ra (*p* < 0.01) were observed in the secreted milieu of the ‘Injured’ MSCs compared with MNCs alone. Similarly, the milieu of the ‘Injured’ MSCs had significantly diminished levels of the proinflammatory IL-12 (*p* < 0.05) and the anti-inflammatory IL-10 (*p* < 0.05) compared with ‘Uninjured’ MSCs. Both IL-1ra and IL-10 have been shown to play a central role in mediating the resolution of ARDS by MSCs [24, 25]. Therefore, decreased secretions of these key anti-inflammatory cytokines may impact MSC potency or their overall capacity to resolve ARDS in vivo. Importantly, however, the fact that all MSCs were able to suppress the secretion of TNF-α in vitro indicates that their anti-inflammatory function is still preserved in spite of injury.

By and large, our results are in line with those reported in the literature. As previously mentioned, Silva et al. performed a similar study in mice. In their study, they evaluated bone marrow cells (not MSCs) and ARDS was induced via LPS administration. Clearly, the type of disease/injury and its sequelae will play a critical role in defining its effects on MSC fate. To our knowledge, ours is the first study that provides a comprehensive evaluation of the characteristics and function of MSCs following ARDS due to smoke inhalation and large surface area burns in large animals. This type of injury is very similar to that seen in burn clinics, especially, but certainly not exclusively, in casualties injured from large explosions on the battlefield. Table 1 summarizes the functional properties of bone marrow MSCs in response to ARDS.

**Table 1:** Functional properties of MSCs from the three different groups

	Uninjured	Injured Untreated	Injured Treated
MSC markers	+++	+++	+++
Differentiation	+++	+++	+++
Clonogenicity	+	+++	+
Proliferation	+++	+++	+
Metabolism	+++	+++	+
Secretion: MSCs	+++	+	+
Gene expression	+++	+	++
Secretion: MSCs + MNCs	+++	+	+

Mesenchymal stem cell (MSC) markers are for CD29^+^, CD90^+^, CD105^+^, and CD45^−^

MSC differentiation is for osteoblasts, adipocytes, and chondrocytes

MSC secretion is in reponse to LPS for IL-1β, IL-6, IL-8, IL-10, and TNF-α

Gene expression is for HMGB1, TLR-4, SOX2, ANG-1, and VEGF

MSC + mononuclear cell (MNC) secretion is for GM-CSF, IFN-γ, IL-1α, IL-1β, IL-1ra, IL-2, IL-4, IL-6, IL-8, IL-10, IL-12, IL-18, and TNF-α

+++ high levels, ++ intermediate levels, + low levels

This study has several limitations. First, the harvested MSCs were expanded in culture; therefore, changes in cell phenotype or function may have been masked due to successive population doublings in vitro. To circumvent this, we have used MSCs at low passages (P2–P3) to achieve the required cell numbers to run the study; however, we expect that at lower population doublings the observed effects due to ARDS may have been more pronounced. Next, since the MSCs were harvested from swine, extrapolation to human MSCs should be done with caution. Although porcine MSCs are highly comparable to human MSCs in terms of their phenotype [26], and swine share similar anatomic and physiologic characteristics with humans [27], interspecies variations in bone marrow should be taken into consideration [28]. Lastly, our study groups included only three animals per group. Therefore, conclusions must be drawn taking into consideration this small sample size.

## Conclusions

In this study, we evaluated the effect of ARDS on MSCs. The results suggest that, following ARDS, there is an increase in the clonogenic capacity of MSCs to increase the available stem cell pool in vivo. Despite the injury, MSCs isolated from ‘Injured’ swine exhibited similar expression levels of common MSCs surface markers as well as maintaining their multipotent differentiation capacity and anti-inflammatory properties. However, MSCs from swine with ARDS exhibited altered characteristics exemplified by downregulation of key genes, diminished secretory potency, and partially compromised immunomodulatory capacity, in which TLR-4 may play a pivotal role. In-vivo administration is necessary to determine whether these ‘Injured’ MSCs are therapeutically inferior to healthy MSCs. These data are important for autologous stem cell-based therapy.

## Abbreviations

AML: Acute myeloid leukemia
Ang-1: Angiopoietin 1
ANOVA: Analysis of variance
ARDS: Acute respiratory distress syndrome
CCM: Complete culture medium
CD: Cluster of differentiation
CFU-F: Colony-forming unit fibroblast
Ct: Threshold cycle
GM-CSF: Granulocyte-macrophage colony stimulating factor
HMGB1: High-mobility group box 1
IFN: Interferon
IL: Interleukin
LPS: Lipopolysaccharide
MNC: Mononuclear cell
MSC: Mesenchymal stem cell
qRT-PCR: Quantitative real-time polymerase chain reaction
SOX-2: Sex determining region Y-box 2
TLR: Toll-like receptor
TNF: Tumor necrosis factor
VEGF: Vascular endothelial growth factor
